# Detection of HIV-1 p24 at Attomole Level by Ultrasensitive ELISA with Thio-NAD Cycling

**DOI:** 10.1371/journal.pone.0131319

**Published:** 2015-06-22

**Authors:** Akira Nakatsuma, Mugiho Kaneda, Hiromi Kodama, Mika Morikawa, Satoshi Watabe, Kazunari Nakaishi, Masakane Yamashita, Teruki Yoshimura, Toshiaki Miura, Masaki Ninomiya, Etsuro Ito

**Affiliations:** 1 Kagawa School of Pharmaceutical Sciences, Tokushima Bunri University, Sanuki, Japan; 2 TAUNS Laboratories, Inc., Izunokuni, Japan; 3 BL Co., Ltd., Numazu, Japan; 4 Faculty of Science, Hokkaido University, Sapporo, Japan; 5 Faculty of Pharmaceutical Sciences, Health Sciences University of Hokkaido, Ishikari-Tobetsu, Japan; 6 Graduate School of Pharmaceutical Sciences, Hokkaido University, Sapporo, Japan; Institut National de la Santé et de la Recherche Médicale, FRANCE

## Abstract

To reduce the window period between HIV-1 infection and the ability to diagnose it, a fourth-generation immunoassay including the detection of HIV-1 p24 antigen has been developed. However, because the commercially available systems for this assay use special, high-cost instruments to measure, for example, chemiluminescence, it is performed only by diagnostics companies and hub hospitals. To overcome this limitation, we applied an ultrasensitive ELISA coupled with a thio-NAD cycling, which is based on a usual enzyme immunoassay without special instruments, to detect HIV-1 p24. The p24 detection limit by our ultrasensitive ELISA was 0.0065 IU/assay (i.e., *ca*. 10^-18^ moles/assay). Because HIV-1 p24 antigen is thought to be present in the virion in much greater numbers than viral RNA copies, the value of 10^-18^ moles of the p24/assay corresponds to *ca*. 10^3^ copies of the HIV-1 RNA/assay. That is, our ultrasensitive ELISA is chasing the detection limit (10^2^ copies/assay) obtained by PCR-based nucleic acid testing (NAT) with a margin of only one different order. Further, the detection limit by our ultrasensitive ELISA is less than that mandated for a CE-marked HIV antigen/antibody assay. An additional recovery test using blood supported the reliability of our ultrasensitive ELISA.

## Introduction

During the window period between infection with human immunodeficiency virus type 1 (HIV-1) and the appearance of detectable antibodies to HIV-1, the infection cannot be diagnosed. Attempts to shorten this period have been made using a fourth-generation immunoassay that detects both HIV-1/2 IgG/M and HIV-1 p24 antigens [1, 2]. However, most of the commercially available detection systems for fourth-generation immunoassays use chemiluminescent measurement and thus need special, high-cost, automated measurement instruments. For this reason, fourth-generation immunoassays are performed only at diagnostics companies and hub hospitals. To overcome this limitation and to test many samples simultaneously, it is important to increase the immunosensitivity of HIV-1 p24 antigen, based on the common enzyme immunoassay without any use of special instruments.

For example, in 2010 French health authorities mandated a limit of detection of at least 2 IU/mL of HIV-1 p24 antigen for a CE-marked HIV antigen/antibody assay (CE is short for Conformité Européenne, i.e., European Conformity) [3, 4]. According to this mandate, commercially available assay kits have been manufactured to detect p24 antigen with the limits of detection ranging from 0.505 to 1.901 IU/mL and from 11.9 to 33.5 pg/mL [4]. The unit of IU/mL is used for the WHO standard, whereas pg/mL is used for the French SFTS standard (i.e., recombinant proteins). Because 1 IU/mL is estimated as 10 pg/mL [5] and MW = 24000 for p24, the best sensitivity in these kits is 0.505 IU/mL, which is *ca*. 2 x 10^−16^ moles/mL.

Thus far, a lot of methods of detecting p24 antigen have been proposed. For example, an immune complex transfer enzyme immunoassay showed a limit of detection of 0.24 pg/mL (i.e., *ca*. 1 x 10^−17^ moles/mL) [6, 7]. A cytometric bead assay was developed to detect p24 at 0.4 pg/mL (i.e., *ca*. 2 x 10^−17^ moles/mL) [8]. A single-molecule immunosorbent assay detected p24 at 0.1 pg/mL (i.e., *ca*. 4 x 10^−18^ moles/mL) [9]. Although a capacitive immunosensor was reported to be marvelously capable of detecting p24 at 7.9 x 10^−8^ pg/mL (i.e., *ca*. 2 molecules/mL), this sensor’s limit of detection in human plasma was substantially greater, at 0.12 pg/mL (i.e., *ca*. 5 x 10^−18^ moles/mL) [10]. Recently, a microchip Europium nanoparticle immunoassay detected 5 pg/mL (i.e., *ca*. 2 x 10^−16^ moles/mL) [11]. In any case, the limit of detection of p24 antigen is not expected to overcome the sensitivity of 10^−17^ to 10^−18^ moles/mL.

However, we have to note HIV testing of many samples requires not only ultrasensitive HIV-1 p24 detection but also rapidity, simplicity, a reasonable cost, and no need for special instruments. Recently, Watabe and colleagues developed an ultrasensitive enzyme-linked immunosorbent assay (ELISA) to determine trace amounts of proteins by combining a conventional ELISA with a thionicotinamide-adenine dinucleotide (thio-NAD) cycling [12]. They claimed that proteins cannot be amplified like nucleic acids by polymerase chain reaction, but that a detectable signal for proteins can be amplified.

**Fig 1:**
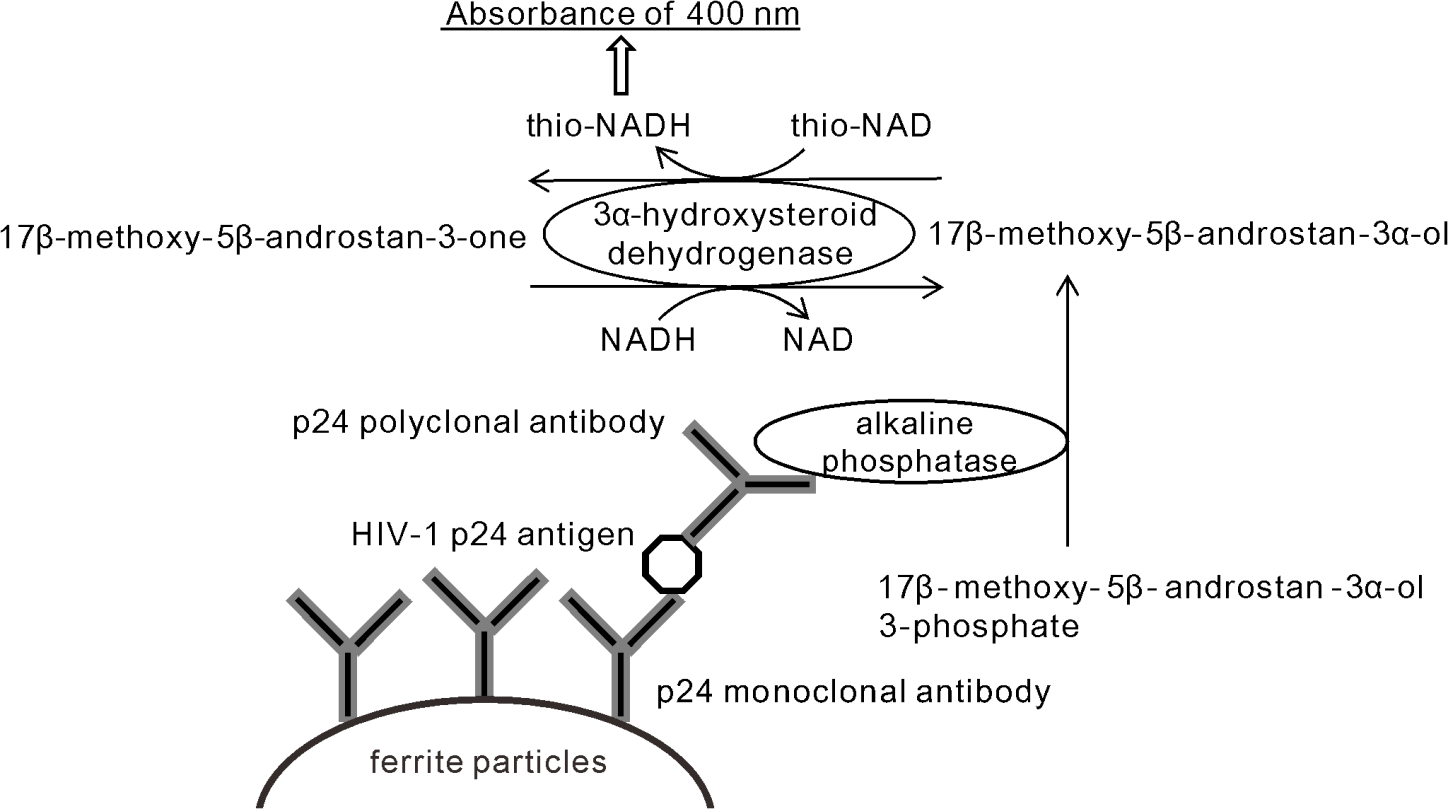
Ultrasensitive detection of HIV-1 p24 antigen by ELISA coupled with a thio-NAD cycling using alkaline phosphatase, androsterone derivatives, 3α-hydroxysteroid dehydrogenase, and its coenzymes.

In their ultrasensitive ELISA (Fig 1), they used 17β-methoxy-5β-androstan-3-ol 3-phosphate as the substrate for alkaline phosphatase (ALP, EC. 3.1.3.1) linked to a secondary antibody, because it is easily hydrolyzed by ALP to 17β-methoxy-5β-androstan-3-ol, which shows a highly efficient thio-NAD cycling. In the thio-NAD cycling, 17β-methoxy-5β-androstan-3-ol is oxidized to 17β-methoxy-5β-androstan-3-one under a catalytic reaction of 3-hydroxysteroid dehydrogenase (3-HSD, EC. 1.1.1.50) with a cofactor thio-NAD [13–17]. By the opposite reaction, 17β-methoxy-5β-androstan-3-one is reduced to 17β-methoxy-5β-androstan-3-ol with a cofactor NADH. During this cycling reaction, thio-NADH accumulated in a quadratic function-like fashion.

The accumulated thio-NADH can be measured directly at an absorbance of 400 nm without any interference from other cofactors. This enables the detection of a target protein with ultrasensitivity (10^−19^ moles/assay) by measuring the cumulative quantity of thio-NADH by a colorimetric method without any use of special instruments for measurements of fluorescence, luminescence or radio isotopes. Further, we should note that their ultrasensitive determination of proteins allows for the detection of trace amounts of proteins only by the application of thio-NAD cycling reagents to the conventional ELISA system. In the present study, we therefore applied this ultrasensitive ELISA to the detection of HIV-1 p24 antigen in blood.

## Materials and Methods

### Chemicals

An HIV-1 p24 antigen that was a WHO International Standard distributed by the National Institute for Biological Standards and Control (NIBSC), NIBSC code (90/636), was purchased from Health Care Technology Foundation (Tokyo, Japan). This antigen was stocked at -20°C as a 1000 IU/mL solution in distilled water. Before use, it was diluted to 0.1–100 IU/mL with TBS including 0.1% BSA. We used only one HIV-1 p24 source in the present study because of the following reason. If the antigen references are different, the data of clinical laboratory examinations may be different. Such circumstances give rise to confusions. The Common Technical Specifications of the European Union published in 2009 thus have required ‘the use of the WHO standard’ (HIV-1 isolate, NIBSC code 90/636; See the following URL. http://eur-lex.europa.eu/legal-content/EN/TXT/PDF/?uri=CELEX:32009D0108&from=EN) [18]. This NIBSC code 90/636 is widely used in clinical laboratory examinations as well as by the medical science researchers.

The primary and secondary antibodies for anti-HIV-1 p24 were derived from an HIV Ag/Ab kit (Lumipulse Presto, Fujirebio, Tokyo, Japan). This kit is widely used as a fourth-generation immunoassay for clinical laboratory examinations, and the antibodies are known to detect HIV-1 p24 antigen in patient blood. The primary antibody consisted of two kinds of monoclonal mouse antibodies (N1-9, N3-3) that were bound with ferrite particles. The secondary antibody was a polyclonal rabbit antibody that linked to alkaline phosphatase (ALP, EC. 3.1.3.1). Thio-NAD and NADH were purchased from Roche (Mannheim, Germany). 3-Hydroxysteroid dehydrogenase (3-HSD; origin from *Comamonas testosteroni*, recombinant by *E*.*coli*) was purchased from Kikkoman Biochemifa (Tokyo, Japan) and purified by BL (Numazu, Japan). 5β-Androsterone was purchased from Steraloids (Newport, RI, USA). 17β-Methoxy-5β-androstan-3-ol 3-phosphate was synthesized as described previously [12]. Absorption was measured (405 nm) with a Corona Electric MTP-500 microplate reader (Hitachinaka, Japan) thermostated at 37°C.

### Ultrasensitive ELISA coupled with thio-NAD cycling

To block nonspecific binding sites, 96-well microplates were incubated with 150 μL of TBS including 1% BSA for 60 min at room temperature, and then washed three times with TBS including 0.05% Tween 20. The primary antibody solution was added at 50 μL per well. Immediately, HIV-1 p24 antigen was added at 50 μL per well, whereas TBS including 0.1% BSA was added as a blank value. The microplates were shaken for 60 min at room temperature, and then washed three times with TBS including 0.05% Tween 20. The secondary antibody solution was added at 50 μL per well, and the microplates were kept for 60 min at room temperature or overnight at 4°C. The microplates were washed six times with TBS including 0.05% Tween 20. To amplify the ELISA signals, a thio-NAD cycling solution of 50 L was added to each well. This solution contains 1.5 mM NADH, 1.5 mM thio-NAD, 0.25 mM 17β-methoxy-5β-androstan-3-ol 3-phosphate, and 40 U/mL 3α-hydroxysteroid dehydrogenase in 50 mM Tris-HCl (pH 8.8). Absorbance at 405 nm was measured with a microplate reader every 15 min for 90 min at 37°C.

### Additional recovery test

All the experimental procedures were the same as the above ultrasensitive ELISA experiments without the antigen solution. We prepared four wells (Table 1) containing:

1. 50 μL of TBS including 0.1% BSA. This was used for a blank experiment.
2. 25 μL of TBS including 0.1% BSA and 25 μL of human control serum (QC-RE Type B Low, Eiken Chemical, Tochigi, Japan). This was also used for a blank experiment.
3. 50 μL of p24 antigen at a concentration of 0.025 IU/50 μL or 0.05 IU/50 μL. This was used for an additional recovery experiment.
4. 25 μL of p24 antigen at a concentration of 0.025 IU/25 μL or 0.05 IU/25 μL and 25 μL of human control serum (QC-RE Type B Low, Eiken Chemical, Tochigi, Japan). This was also used for an additional recovery experiment.

**Table 1:** Protocol for additional recovery test.

	blank		sample	
solution	(1)	(2)	(3)	(4)
TBS including 0.1% BSA (L/well)	50	25	50	25
Human control serum (L/well)	0	25	0	25
HIV-1 p24 antigen (0.025 or 0.05 IU/well)	‒	‒	+	+

The additional recovery ratio was calculated by [the absorbance of (4) ‒ that of (2)]/[the absorbance of (3) ‒ that of (1)].

### Chemiluminescent enzyme immunoassay as a control experiment

The chemiluminescent enzyme immunoassay was performed by the protocols established by the manufacturer of an HIV Ag/Ab kit (Lumipulse Presto, Fujirebio). Briefly, the basic ELISA experiments were the same as our colorimetric ultrasensitive ELISA. However, instead of amplification of the ELISA signals by a thio-NAD cycling, the chemiluminescent substrate solution including AMPPD (3-(2’-spiroadamantane)-4-methoxy-4-(3”-phosphoryloxy)phenyl-1,2-dioxetane disodium salt) was added at 200 L per well and incubated for 4 min at 37°C. Then, the luminescence of 477 nm was measured with a multiplate reader (POLARStar OPTIMA, BMG Labtech, Ortenberg, Germany).

### Limit of detection, limit of determination, and coefficient of variation

The experimental data were obtained by subtracting the mean value of the blank signals from each of the corresponding measured data. The limit of detection was estimated from the mean of the blank, the standard deviation of the blank, and a confidence factor of 3. The limit of determination was estimated by the same method as for the limit of detection, but with the use of a confidence factor of 10. The coefficient of variation calculated from 4–5 data points was obtained for 1.0 IU/mL of p24.

## Results

**Fig 2:**
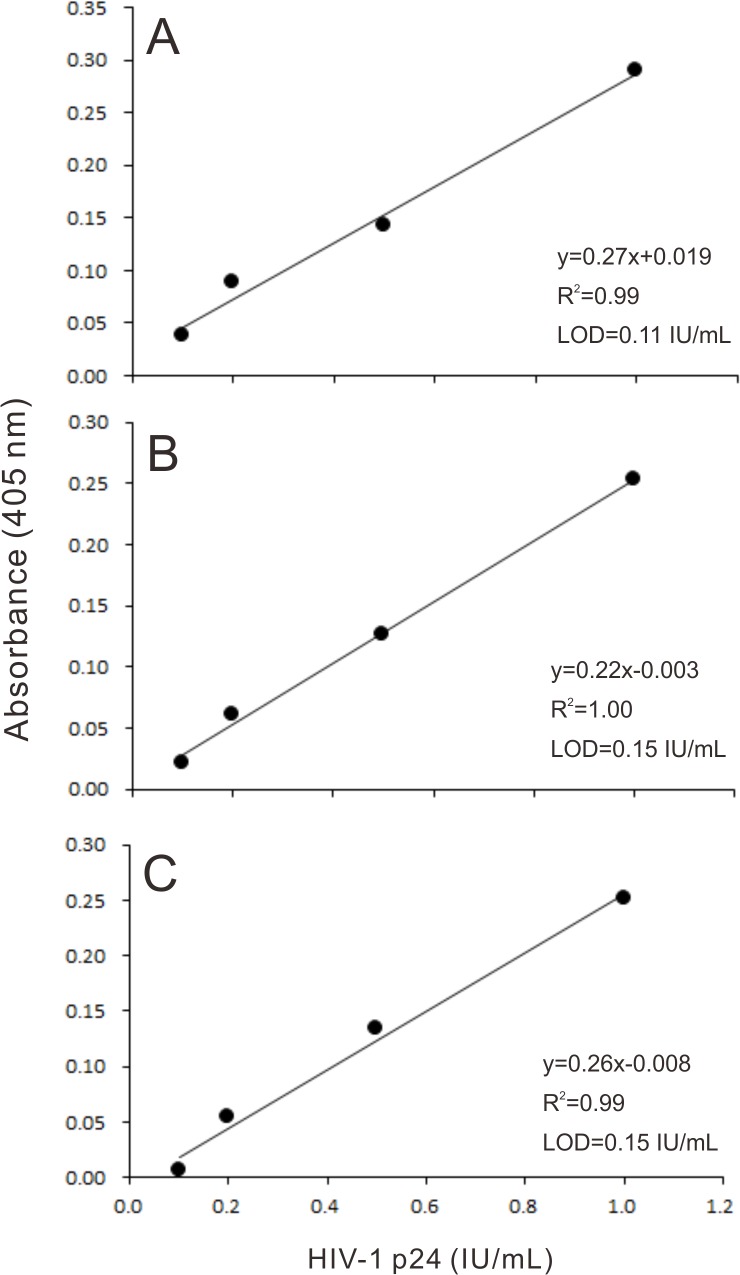
Linear calibration curves for HIV-1 p24 antigen obtained by an ultrasensitive ELISA coupled with a thio-NAD cycling. The absorbance of thio-NADH was measured at a cycling reaction of 90 min. Three data measured in different plates are presented as A, B and C. LOD is short for the limit of detection.

We obtained three linear calibration curves for HIV-1 p24 antigen in the range of 0.1 ‒ 1.0 IU/mL that were provided with the ultrasensitive ELISA coupled with a thio-NAD cycling (Fig 2). The curves were obtained from the absorbance of thio-NADH at a cycling reaction of 90 min. The data were measured in different plates. One linear calibration curve is expressed as *y* = 0.27*x* + 0.019, *R* ^2^ = 0.99 (Fig 2A). The limit of detection of p24 was 0.0055 IU/assay (i.e., *ca*. 2 x 10^−18^ moles/assay). We thus claim that the ultrasensitive ELISA coupled with a thio-NAD cycling succeeds in detecting p24 at the attomole level. The minimum limit of determination of p24 was 0.0275 IU/assay (i.e., *ca*. 1 x 10^−17^ moles/assay).

Another linear calibration curve is expressed as *y* = 0.22*x* ‒ 0.003, *R* ^2^ = 1.00 (Fig 2B). The limit of detection was 0.0075 IU/assay (i.e., *ca*. 3 x 10^−18^ moles/assay). The minimum limit of determination was 0.0240 IU/assay (i.e., *ca*. 1 x 10^−17^ moles/assay). Another linear calibration curve is expressed as *y* = 0.26*x* ‒ 0.008, *R* ^2^ = 0.99 (Fig 2C). The limit of detection was 0.0075 IU/assay (i.e., *ca*. 3 x 10^−18^ moles/assay). The minimum limit of determination of p24 was 0.0210 IU/assay (i.e., *ca*. 9 x 10^−18^ moles/assay).

In our measurement system, one assay contained a 50 L solution, and thus our values of the limit of detection and the minimum limit of determination, which were calculated as a mean value of the above data, corresponded to 0.13 ± 0.02 and 0.43 ± 0.14 IU/mL (mean ± SD, n = 3), respectively. These are *ca*. 6 x 10^−17^ and 2 x 10^−16^ moles/mL. That is, even in the expression of concentration per mL, our value is less than one‒tenth of the value that the French health authorities require [3, 4]. The coefficient of variation was 8% for 1 IU/mL.

For the additional recovery tests, we tested two concentrations for p24 with the serum. When we used 0.025 IU/well (0.5 IU/mL) p24, the additional recovery ratio was 106.3 ± 7.1% (mean ± SD, n = 3), and when we used 0.05 IU/well (1.0 IU/mL) p24, the additional recovery ratio was 101.0 ± 11.4% (mean ± SD, n = 3). That is, the additional recovery test showed that our ultrasensitive ELISA can detect p24 in blood.

For comparison of our system with a commercially available detection system, a chemiluminescent enzyme immunoassay (Lumipulse Presto, Fujirebio) was used. This assay was not colorimetric, and thus it is expensive and requires a special instrument. For this assay, a linear calibration curve (*y* = 1038*x* ‒ 431, *R* ^2^ = 0.99; note that this curve were obtained by not subtracting the mean value of blank signals at 0 min because of the too-fast reaction of AMPPD.) was obtained for HIV-1 p24 in the range of 1 ‒ 10 IU/mL. This curve was obtained from the luminescence of AMPPD at 4 min. The limit of detection of p24 that we obtained was 0.27 IU/mL (i.e., *ca*. 1 x 10^−16^ moles/mL), whereas that of the package insert data of the manufacturer indicates 0.7 IU/mL. The minimum limit of determination of p24 that we obtained was 0.92 IU/mL (i.e., *ca*. 4 x 10^−16^ moles/mL). To our knowledge, this chemiluminescent enzyme immunoassay (Lumipulse Presto, Fujirebio) shows the best sensitivity for p24 in commercially available detection systems in Japan at present. That is, our colorimetric ultrasensitive ELISA shows better sensitivity to some extent than the chemiluminescent enzyme immunoassays, and our system provides some overwhelming advantages in cost and simplicity without the need for a special instrument.

## Discussion

It is important to diagnose primary HIV-1 infection and begin antiretroviral treatment as early as possible. Most HIV-1/2 antibody diagnostic tests detect the antibodies for the antigens of HIV-1 gp41 and HIV-2 gp36, which are highly conservative transmembrane proteins. These tests are quick and easy, and thus have been widely used in many clinics and public health centers. However, when only the antibody diagnostic tests are used, there is a long delay (22 days of window period, for example) before diagnosis is possible [19]. To shorten this delay, HIV-1 p24 antigen, which is expected to increase in number before antibodies emerge, should be detectable in trace amounts. Generally, the gold standard for diagnosing HIV-1 is PCR-based nucleic acid testing (NAT) [19], but this is expensive and has infrastructure requirements, a long measuring time, and high complexity, thereby limiting its usefulness for large numbers of samples. The ultrasensitive detection of HIV-1 p24 antigen for early diagnosis is thought to be a simple and reasonable alternative to NAT for monitoring treatment and protecting the blood supply [20].

As described above, HIV-1 p24 in blood emerges transiently at the very early period after infection, and its concentration returns to the basal level quickly (for example, see the following URL. http://www.mayomedicallaboratories.com/articles/hottopics/transcripts/2009/2009-10a-hiv/10a-2.html). An HIV-1 p24 test is, therefore, very useful as a screening in the early stage of infection, but it is difficult for us to obtain the blood samples from such patients in the very early period after infection. In other words, the patients, who have been diagnosed as HIV infection by commercially available methods, are not suitable for our HIV-1 p24 tests. To overcome these practical problems, we attempted to perform the additional recovery tests in which the HIV-1 p24 antigen was added into the control serum. Because our results demonstrated that the ratio was about 100% for 0.5 IU/mL of HIV-1 p24, which was less than the value (2 IU/mL) required for a CE-marked HIV antigen/antibody assay (see Introduction), our ultrasensitive method is sufficiently thought to detect HIV-1 p24 antigen in human blood obtained from the patients in the very early period after infection.

We want to reconsider whether NAT is really the gold standard for diagnosing HIV-1. Barletta et al. claimed that the target protein (i.e., HIV-1 p24 antigen) is present in the virion at much higher numbers than viral RNA copies (approximately 3000 HIV-1 p24 antigen molecules vs. 2 RNA copies per virion) [21]. The 10^−18^ moles/assay value in our present results corresponds to 10^6^ protein molecules/assay. This corresponds to *ca*. 10^3^ RNA copies/assay. Although in laboratory conditions a real-time PCR (i.e., NAT) can detect 10^1^-order RNA copies/assay [18, 22], the limitation of detection is usually 10^2^-order RNA copies/assay [23]. That is, our ultrasensitive ELISA coupled with a thio-NAD cycling for HIV-1 p24 is chasing the data obtained by NAT with a margin of only one different order.

Previously, Watabe et al. succeeded in detecting ALP at 10^−20^ moles/assay by a thio-NAD cycling reaction of the combination of 3-hydroxysteroid 3-phosphate, 3-HSD, thio-NAD, and NADH [12]. Thus, our method will hopefully be capable of detecting p24 with sufficient sensitivity to overcome the value obtained with NAT, when much more suitable antibodies become available.

The CV value (8%) obtained from our experiments seems reasonable. Miedouge and colleagues have reported that the CV’s obtained from five different HIV-1 p24 antigen tests, all of which were commercially available, using a WHO standard were 1.5, 4.8, 5.4, 9.0, and 10.2%, respectively [3]. These data also support that our ultrasensitive ELISA for HIV-1 p24 is at a level high enough for practical use.

Recently, the detection level, but not the determination level, of HIV-1 p24 is now advanced to 1 x 10^−18^ g/mL (i.e., *ca*. 2 x 10^−23^ moles/mL) by nanotechnology [24]. This is an extraordinary sensitivity, and this method has the advantage of being detectable with the naked eye. However, the specificity and stability of the reactions and the usefulness should be carefully followed in the future.

## Conclusions

Our ultrasensitive ELISA coupled with a thio-NAD cycling is very convenient because it requires only the addition of a thio-NAD cycling solution to the usual ELISA without any use of special instruments. Consequently, the present method reported here can be widely used as a powerful tool to test many samples simultaneously.
